# Dinutuximab beta plus conventional chemotherapy for relapsed/refractory high-risk neuroblastoma: A single-center experience

**DOI:** 10.3389/fonc.2022.1041443

**Published:** 2022-12-23

**Authors:** Nur Olgun, Emre Cecen, Dilek Ince, Deniz Kizmazoglu, Birsen Baysal, Ayse Onal, Ozhan Ozdogan, Handan Guleryuz, Riza Cetingoz, Ayse Demiral, Mustafa Olguner, Ahmet Celik, Serra Kamer, Erdener Ozer, Zekiye Altun, Safiye Aktas

**Affiliations:** ^1^ Department of Pediatric Oncology, Dokuz Eylul University Institute of Oncology, Izmir, Türkiye; ^2^ Department of Nuclear Medicine, Dokuz Eylul University School of Medicine, Izmir, Türkiye; ^3^ Department of Radiology, Dokuz Eylul University School of Medicine, Izmir, Türkiye; ^4^ Department of Radiation Oncology, Dokuz Eylul University School of Medicine, Izmir, Türkiye; ^5^ Department of Pediatric Surgery, Dokuz Eylul University School of Medicine, Izmir, Türkiye; ^6^ Department of Pediatric Surgery, Ege University School of Medicine, Izmir, Türkiye; ^7^ Department of Radiation Oncology, Ege University School of Medicine, Izmir, Türkiye; ^8^ Department of Pathology, Dokuz Eylul University School of Medicine, Izmir, Türkiye; ^9^ Department of Basic Oncology, Dokuz Eylul University Institute of Oncology, Izmir, Türkiye

**Keywords:** neuroblastoma, relapsed, refractory, dinutuximab beta, anti-GD2

## Abstract

**Background:**

Relapsed/refractory high-risk neuroblastoma has a dismal prognosis. Anti-GD2-mediated chemo-immunotherapy has a notable anti-tumor activity in patients with relapsed/refractory high-risk neuroblastoma. The purpose of this study was to analyze the efficacy and safety of the combination of immunotherapy with dinutuximab beta (DB) and chemotherapy in patients with relapsed/refractory high-risk neuroblastoma.

**Methods:**

All patients received the Turkish Pediatric Oncology Group NB 2009 national protocol for HR-NB treatment at the time of diagnosis. Salvage treatments were administered after progression or relapse. The patients who could not achieve remission in primary or metastatic sites were included in the study. The most common chemotherapy scheme was irinotecan and temozolomide. DB was administered intravenously for 10 days through continuous infusion with 10 mg/m^2^ per day. The patients received 2 to 14 successive cycles with duration of 28 days each. Disease assessment was performed after cycles 2, 4, and 6 and every 2 to 3 cycles thereafter.

**Results:**

Between January 2020 and March 2022, nineteen patients received a total of 125 cycles of DB and chemotherapy. Objective responses were achieved in 12/19 (63%) patients, including complete remission in 6/19 and partial response in 6/19. Stable disease was observed in two patients. The remaining five patients developed bone/bone marrow and soft tissue progression after 2-4 cycles of treatment. The most common Grade ≥3 toxicities were leukopenia, thrombocytopenia, hypertransaminasemia, fever, rash/itching and capillary leak syndrome, respectively.

**Conclusion:**

Our study results suggest that DB-based chemo-immunotherapy seems to be suitable with encouraging response rates in patients with relapsed/refractory high-risk neuroblastoma.

## Introduction

Neuroblastoma (NB), which is the most common extracranial solid tumor of childhood, presents with high-risk (HR) disease in nearly half of the cases at the initial diagnosis. Before the introduction of immunotherapy, long-term remission could be achieved in about 40% of HR patients with multimodal treatment including induction chemotherapy, surgery, myeloablative chemotherapy with autologous stem cell rescue, radiation and maintenance therapy with isotretinoin (1–3). Anti-disialoganglioside 2 (anti-GD2)-based immunotherapy has altered the perspective for patients with high-risk NB (4).

The contribution of the anti-GD2 antibody dinutuximab (Unituxin^®^) to the standard treatment has healed outcomes with a clear survival benefit (up to >60%) in patients with HR NB in the COG-ANBL0032 trial, which led to the ratification of this drug in the United States (5, 6). Recently, a similar anti-GD2 antibody, dinutuximab beta (DB) (Qarziba^®^), has been added to the current standard of care for patients with HR NB in Europe, according to the results of the International Society of Paediatric Oncology European Neuroblastoma (SIOPEN) trials (7, 8). Dinutuximab beta is a chimeric human/mouse monoclonal IgG1 antibody produced in the CHO (Chinese hamster ovary) mammalian cell line using recombinant deoxyribonucleic acid (DNA) technology (9). It is directed against the GD2 disialoganglioside, which has limited expression in normal tissues, but is highly expressed across several tumor entities including NB. The European Medicines Agency (EMA) approved the agent in 2017 for the treatment of HR-NB in patients aged ≥12 months who achieved at least a partial response (PR) to induction chemotherapy and received myeloablative therapy and stem cell transplant and patients with a history of R/R HR-NB (9, 10). However, this agent was not available in Turkey until two years ago. Recently, it was included in the reimbursement list of the Republic of Turkey, Ministry of Health, Social Security Institution and it is currently available for the treatment of patients with HR-NB.

In the present study, we purposed to investigate the impacts and adverse effects of the combination of immunotherapy with DB and chemotherapy in patients with R/R HR-NB.

## Materials and methods

### Study design

This study was managed at the Institute of Oncology, Department of Pediatric Oncology center between January 2020 and March 2022. A written informed consent was obtained from each parent and/or legal guardians. The study protocol was approved by the institutional Ethics Committee. The study was conducted in accordance with the principles of the Declaration of Helsinki.

Patients of over 12 months with documentation of an HR-NB diagnosis were eligible at relapse or designation of refractory disease status. Inclusion criteria were as follows: relapsed or refractory, measurable by contrast-enhanced magnetic resonance imaging (MRI) and/or computed tomography (CT) or metaiodobenzylguanidine (mIBG)/fluorodeoxyglucose (FDG) positron emission tomography (PET)/CT (if non-MIBG-avid lesions were present at study enrollment) evaluable disease and/or demonstrated by bone marrow aspiration and biopsy. All patients were previously treated with the 2009 Turkish Pediatric Oncology Group NB national protocol at the time of diagnosis. (dose-intensive induction chemotherapy ± surgery ± radiotherapy or high-dose chemotherapy (HDCT), followed by autologous stem cell transplantation [ASCT]). In the TPOG National NB protocol, high-risk patients were treated in one of the two treatment arms (conventional chemotherapy arm and HDCT & ASCT arm), which were not randomized and decided according to the physical conditions of the centers and the discretion of the physician. Tandem application was not performed in the HDCT+ASCT arm of the TPOG NB high-risk group.

Salvage treatments were administered after progression or relapse including ICE (ifosfamide + carboplatin + etoposide), TVD (topotecan + vincristine + doxorubicin) or TVC (topotecan + vincristine + cyclophosphamide) ± temsirolimus ± bevacizumab, RIST (rapamycin, irinotecan, sunitinib, temozolomide), and IT (irinotecan–temozolomide). Patients with bone marrow as the only site of disease were excluded from the study.

### Treatment protocol and procedures

The most common chemotherapy scheme was irinotecan (IV, 50 mg/m² per dose, on Days 0-4) and temozolomide (PO, 100 mg/m² per dose, on Days 0-4). Vincristine + topotecan + temozolomide, topotecan + etoposide + temozolomide, carboplatin + etoposide + temozolomide and ICE were the other chemotherapy regimens applied. Dinutuximab beta (Qarziba^®^, EUSA Pharma) was administered intravenously for 10 days through continuous infusion with 10 mg/m^2^ per day (on Days 1-10). The patients received 2 to 14 successive cycles for a total of 28 days. Pain controlling consisted of gabapentin (PO, 15 mg/kg/day in three doses) starting three days prior to start of DB infusion, and acetaminophen (PO, 60 mg/kg/day in four doses, with a maximum of 4 g/day) and morphine (IV, 10 μg/kg/h) starting 1 and 2 h before the start of DB infusion, respectively. Prophylactic cefixime was used to reduce irinotecan-induced diarrhea. Loperamide was administered to patients with diarrhea. On-therapy patients were evaluated for renal, hepatic, and hematological functions every other day. Transfusion was performed to ensure a hemoglobin level of >8 g/dL, platelet count of >75,000/mm^3^, and albumin of >3.5 g/dL. Side effects were graded according to the NCI Common Terminology Criteria for Adverse Events (CTCAE) (version 5.0) (11).

### Outcome measures and definitions

All patients were assessed within two weeks of study enrollment. The response was assessed after 2, 4, and 6 cycles and every 2 to 3 cycles thereafter. Response was evaluated using the revised International Neuroblastoma Response Criteria (INRC) for disease assessment (12). For mIBG evaluation, soft tissue and skeletal response was assessed using the SIOPEN scoring method (13). Bone marrow involvement was evaluated using routine staining and bilateral malignant cell rate was identified. All imaging and histopathological specimens were reviewed by a single pediatric radiologist, nuclear medicine experts, and a pediatric pathologist.

## Results

A total of 19 patients received a total of 125 cycles of DB+CT. The median age at the time of study enrollment was 5.5 (range, 2.5 to 11) years. The median follow-up was 11 (range, 6 to 26) months. All patients had INRG Stage M disease and one had central nervous system disease (Patient No. 4, the patient’s CNS disease has been clinically stable for six months prior to starting this study and assessment was made clinically and by CT or MRI) at the time of diagnosis. While the NMYC amplification was detected in three patients at the time of diagnosis, it was absent in 14 patients. The NMYC amplification status was unknown in the remaining two patients. At the time of study enrollment, all patients, except for one, had bone metastasis, while 13 had bone marrow metastasis, three had dural involvement, and eight had a soft tissue mass. Ten of the patients were in relapse and nine of them were in refractory disease (2/9 patients had progressive disease) at the time of enrollment in the study. None of the patients had only bone marrow involvement. Three (37%) of our patients were treated in the high-dose chemotherapy and ASCT arm before DB plus conventional chemotherapy. One patient previously received three cycles of DB maintenance therapy (**Table 1**).

**Table 1:** Characteristics of Patients.

CaseNo	Age at Diagnosis (years)	R/R time(Months)	MYCN	Previous treatment	Disease statusat enrolment	Site of diseaseat enrolment	CT regimen+ DB	Totalcycles	SIOPENScores*	INRCstatus	F/U
1	9	4	NA	A9+A11 TVC VIT+Tems TED+Tems	Refractory	Soft tissue Bone Bone marrow	IT TVT ICE	12	64/50	PD	DOD
2	6	3	NA	A9+A11, TVC+ Tems+Bvzm TVD+ Tems+Bvzm RIST	Refractory	Soft tissue Bone Bone marrow	TET	7	8/0	CR	A
3	6	3	NA	A9+A11 ICE, TVC, RIST	Refractory	Bone Bone marrow	IT	6	21/0	CR	A
4	2.5	72	NA	A9+A11 TVC, HDT/ASCT IT, VEC	Refractory	Bone Bone marrow Dural involvement	IT CET	7	5/9	PD	DOD
5	6	48	NA	A9+A11+HDT/ASCT Eflornithine (DFMO)	Relapsed	Bone Bone marrow Liver	IT	14	PET-CT	CR	A
6	3	6	NA	A9+A11 ICE, TVC, RIST	Refractory	Bone Bone marrow	IT	11	53/32	SD	A
7	4	12	AMP	A9+A11+HDT/ASCT ICE	Relapsed	Soft tissue Distant LAP	IT	6	PET-CT	PD	A
8	2	10	UN	A9+A11+HDT/ASCT ICE, IT	Relapsed	Bone	IT	8	PET-CT	CR	A
9	3	20	NA	A9+A11+HDT/ASCT MIBG, TVC	Relapsed	Bone	IT	5	7/1	PR	A
10	3	7	NA	A9+A11	Refractory	Soft tissue Bone Bone marrow	IT	6	41/0	CR	A
11	2	4	UN	A9+A11 TVD	Refractory/ progressive	Soft tissues Bone Bone marrow Dural involvement	IT ICE	2	42/48	PD	DOD
12	3	10	NA	A9+A11+HDT/ASCT	Refractory	Bone	IT	4	5/1	PR	A
13	2	5	AMP	A9+A11+HDT/ASCT	Refractory	Bone Bone marrow	TVD IT ICE	8	40/23	SD	A
14	2	12	NA	A9+A11 TVC VIT, HDT/ASCT	Refractory/ progressive	Soft tissues Bone Bone marrow Dural involvement	IT	3	32/42	PD	DOD
15	4	12	NA	A9+A11+HDT/ASCT	Relapsed	Soft tissue Bone Bone marrow	IT	6	4/3	PR	A
16	5	25	NA	A9+A11+HDT/ASCT MIBG	Relapsed	Bone Bone marrow	IT	7	9/2	PR	A
17	3	26	NA	A9+A11+HDT/ASCT	Relapsed	Soft tissue Bone Bone marrow Dural involvement	IT	3	4/1	PR	A
18	2	29	AMP	A9+A11 DB (3 CYCLES)	Relapsed	Bone	IT	6	2/0	CR	A
19	6	15	NA	A9+A11	Relapsed	Bone	IT	6	5/1	PR	A

*SIOPEN scores; at enrollment/at the end of the last cure. ICE; Ifosfamide + Carboplatin + Etoposide; VEC, Vinblastin + Etoposide + Cisplatin; TVT, Topotecan + Vincristine + Temozolomide; TET, Topotecan + Etoposide + Temozolomide; CET, Carboplatin + Etoposide + Temozolomide; TVC, Topotecan + Vincristine + Cyclophosphamide; RIST, Rapamycin + Irinotecan + Sunitinib + Temozolomide; VIT, Vincristine + Irinotecan + Temozolomide; IT, Irinotecan + Temozolomide; A9; Vincristine + Dacarbazine + Doxorubicin + Ifosfamide; A11, Cyclophosphamide + Etoposide + Cisplatin; TVD, Topotecan + Vincristine + Doxorubicin; HDT/ASCT, High-dose chemotherapy followed by autologous stem cell transplantation; AMP, amplified; NA, Non-amplified; A, Alive; DOD, Death Of Disease, PR, Partial Response; CR, Complete Response; SD, Stable Disease; PD, Progressive Disease; LAP, lymphadenopathy; F/U, follow-up; DB, Dinutuximab beta; R/R time, relapsed/refractory time.

**Figure 1:**
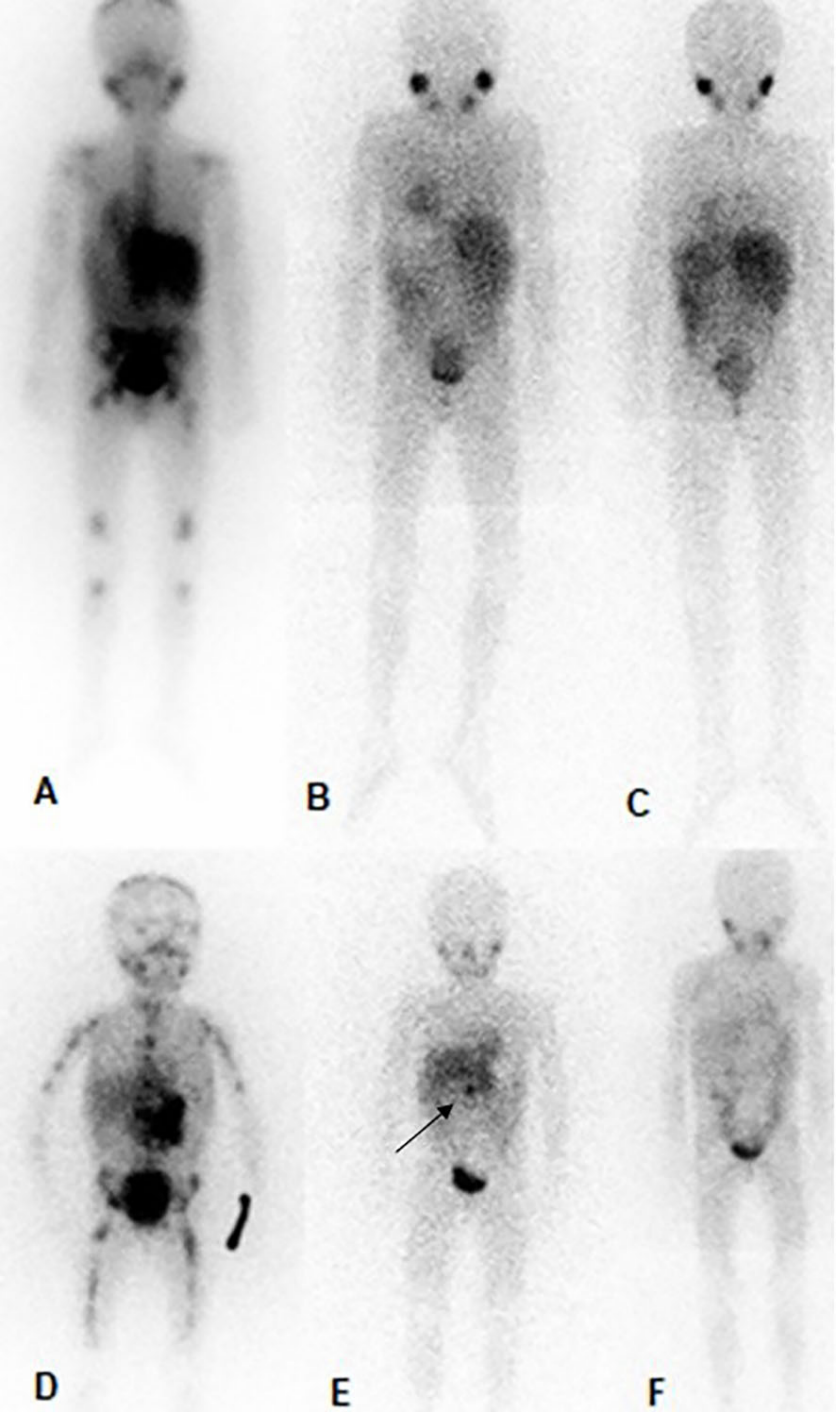
MIBG in patient no. 3 (the above) and patient no. 10 (below). Patient no. 3, **(A)**; prior to therapy with DB and chemotherapy (SIOPEN score of 21), **(B)**; After 6 cycles of treatment complete regression of the skeletal metastases and the soft tissue mass (SIOPEN score of 0), **(C)**; 6 months after the end of DB and chemotherapy (SIOPEN score of 0). Patient no. 10, **(D)**; prior to therapy with DB and chemotherapy (SIOPEN score of 41), **(E)**; After 6 cycles of treatment complete regression of the skeletal metastases and substantial regression the soft tissue mass-primary tumor (SIOPEN score of 1, black arrow), **(F)**; 6 months after the end of DB and chemotherapy (SIOPEN score of 0).

Objective responses (OR; complete response (CR) or partial response (PR)) were achieved in 12 (63%) patients (CR in six (6/19) patients and PR in six (6/19) patients). Four patients with a MIBG score of 0 were considered CR (two of them are shown in **Figure 1**). Three (two were PET-negative and the other was progressive disease) of 19 patients were evaluated with PET/CT, as they had MIBG non-avid disease. No disease was detected in bone marrow biopsy in any of the patients with an objective response. All patients with complete response achieved best response after six cycles. Patients with partial response (n=6) received at least three and at most seven cycles of DB and chemotherapy, and four of these patients had a MIBG score of one in their most recent evaluation. Stable disease (SD) was identified in two (2/19) patients. In one of these (Patient No. 6), the MIBG score regressed from 52 to 32, and the initial bone marrow involvement disappeared. The patient was also evaluated with PET/CT which revealed negative results. Progressive disease (PD) was observed in five (5/19) patients. Four patients died from PD. One patient (Patient No. 1) of non-survivors achieved a CR in bone marrow after cycle five, but she developed PD after the ninth cycle of treatment. Two patients (Patient No. 11 and 14) were non-responsive to treatment and died from PD while starting DB treatment.

None of the patients developed unacceptable toxicities. The most common side effect was fever, which was more common in the first cycles of treatment. Broad-spectrum antibiotics were used after cultures were taken in patients with fever. Other than catheter infection and/or neutropenic sepsis, DB treatment was not discontinued. Diarrhea, tachycardia, pain, nausea/vomiting rash and hypertension were other common side effects (**Table 2**). The dose of morphine was tapered and discontinued in patients who did not have or relieved from pain. Patients with morphine-related side effects such as sedation, constipation, oliguria and urinary retention improved, when morphine was discontinued. The DB infusion rate reduced or infusion was interrupted in patients with severe hypertransaminasemia (persistent increases in transaminase levels to 5xULN (upper limit of normal)). In patients with tachycardia, propranolol was used based on cardiac evaluation findings. Anaphylaxis was not observed in any patient. However, despite adequate premedication, especially in patient 5, an allergic reaction and itching developed (grade 3) that caused the infusion to be stopped in the first 6 cycles. Extravasation was seen in two patients. Infusion was discontinued, and cold application and limb elevation were performed. Symptoms regressed and no sequelae were observed in both patients. Mild hypoxia (≥90% O_2_) was detected in one patient without any other finding such as capillary leak syndrome or anaphylaxis. In patient 6, swelling on the cheek and face was common in almost every cure, and the infusion was not stopped. However, all patients received 100% of cumulative designed course dose of DB, even if the treatment period exceeded 10 days.

**Table 2:** Therapy-related Toxicities.

Adverse Events	Number of Cycles		
	Total (N=125)	Grade 3N (%)	Grade 4N (%)
Leukopenia	105 (84%)	50 (40%)	27 (22%)
Anemia	102 (82%)	0	0
Thrombocytopenia	95 (76%)	20 (16%)	14 (11%)
Hypertransaminasemia	67 (54%)	21 (17%)	10 (8%)
Fever	105 (84%)	17 (14%)	0
Diarrhea	56 (45%)	0	0
Tachycardia	50 (40%)	0	0
Anorexia, nausea, vomiting	48 (38%)	0	0
Pain	44 (35%)	0	0
Rash and itching	37 (30%)	13 (11%)	0
Hypertension	27 (22%)	0	0

## Discussion

Our study results showed that DB-based chemo-immunotherapy is effective and safe in patients with relapsed or refractory (R/R) HR-NB. Despite all advances in NB treatment, relapsed/refractory disease still remains the major obstacle to cure (14–16). To date, several salvage therapies including ifosfamide, carboplatin, and etoposide (ICE) (17); topotecan and cyclophosphamide (18, 19); vincristine, topotecan and cyclophosphamide (TVC) (20); topotecan and cyclophosphamide and etoposide (21); temozolomide and topotecan (TOTEM) (22); irinotecan and temozolomide (with or without bevacizumab) (23–25); topotecan, vincristine, and doxorubicin (TVD) (26, 27), and the high-dose ^131^I-MIBG therapy (28) have been widely used for the treatment of R/R HR-NB; however, the response rates are unsatisfactory. Although anti-GD2 monoclonal antibodies (mAbs) were initially approved to treat minimal residual disease, recent studies with anti-GD2 mAbs have been performed in HR-NB patients with intractable mass of soft tissue or bone/bone marrow disease. An article from the Children’s Oncology Group (COG) including patients with R/R NB treated with irinotecan, temozolomide, dinutuximab, and GM-CSF demonstrated (ANBL1221) considerable objective responses in nine (53%) (including five with complete response) of 17 patients (29). In this study with additional patients, objective responses (OR) were seen in 22 (41.5%) (including 11 with CR) of 53 patients, while stable disease (SD) was observed in 22 of 53 patients (30). Of note, patients with only bone marrow disease were not included in these studies. The overall rate of patients with bone marrow disease was 62.5% (randomly and non-randomly assigned cohort patients) (30). Based on our experience with the combination of DB with irinotecan and temozolomide for patients with evaluable or measurable R/R disease, OR was achieved in 12 (63%) of 19 evaluable patients. None of the patients had bone marrow involvement alone. Bone metastases were present in all patients, except for one (Patient No 7).

Since dinutuximab and DB are similar but different drugs, the administration schemes and doses vary. Dinutuximab is given by combined application with interleukin-2 (IL-2), granulocyte-macrophage colony stimulating factor (GM-CSF), and 13-cis-retinoic acid (RA), whereas DB is used with RA as maintenance therapy and should be combined with IL-2 in patients with a history of relapsed/refractory disease and in patients who have not achieved a complete response after first-line therapy (10, 31). However, further studies have shown no benefit of adding IL-2 to DB treatment in neither HR-NB nor R/R settings, except for a significant increase in toxicity (32). Therefore, we did not use IL-2 in our study. The use of GM-CSF is known to increase anti-GD2 activity, but cannot be used with DB, as it is not commercially available in Europe. To the best of our knowledge, there is no study in the literature in which the results of the use of DB in patients with R/R NB are reported. However, there are ongoing clinical trials investigating the use of DB alone or in combination with other treatment options in patients with R/R NB (33–36). Two [NCT01701479 (34) and NCT02743429 (33)] of these studies did not include patients with progressive disease at the time of enrollment. In our study, two of our patients who had signs of progression at the time of study entry were not excluded. A higher objective response rate is obtained if these patients are not included in the evaluation. Results of the ongoing Phase 1 trial (Minivan) will demonstrate the efficacy of nivolumab (anti-Programmed Cell Death Protein 1 (anti-PD1) antibody) and DB combination after non-myeloablative MIBG therapy in patients with R/R NB. Ehlert et al. (37) described two patients with R/R NB benefited from nivolumab and DB therapy. A complete response was achieved in one patient and a very good partial response was obtained in the other.

Although the role of DB in the maintenance therapy in HR NB is well-documented (7, 8), there is a limited number of data regarding responses in evaluable disease. Federico et al. (38) completed a pilot study using a humanized anti-GD2 mAb (hu14.18K322A) with chemotherapy and natural killer cells in children with recurrent/refractory NB and demonstrated an objective response rate of 61%. According to these results, a phase 2 study was started in which hu14.18K322A and cytokines were combined with induction chemotherapy of newly diagnosed NBL patients. According to this study, adding hu14.18K322A to induction chemotherapy improved early objective responses, significantly reduced tumor volumes in most patients, and improved end-of-induction response rates (39). Gartrell et al. (40) reported 6 patients with newly diagnosed high-risk NB treated by dinutuximab combined with induction chemotherapy, IL-2, and GM-CSF. All patients tolerated and benefited well from induction chemotherapy and dinutuximab therapy. More recently, Spasov et al. (41) noticed positive results with the use of DB in three patients in whom a complete response was unable to be achieved at the end of induction therapy. Additional data from large-scale, multi-center studies and clinical cases are essential to optimize the treatment schedule of anti-GD2 mediated chemo-immunotherapy in HR-NB.

In our study, after 10 cycles of DB and chemotherapy treatment, one patient (Patient No. 6) whose MIBG scores were improved by almost half (53 to 26) with no bone marrow disease and with negative PET/CT result was considered as having stable disease. In a recently reported study, it has been suggested that anti-gd2-induced tumor differentiation may occur in patients with persistent bone lesions on MIBG by performing histopathological examination (42). In this study, naxitamab, a different anti-gd2 monoclonal antibody, was combined with irinotecan, temozolomide and GM-CSF. Although the SIOPEN score of the other patient (Patient No. 13) decreased from 40 to 23, the disease was considered stable because bone marrow involvement continued.

Although DB-related side effects can be difficult to treat and variable, they are usually manageable. Close monitoring of patients by an experienced team, complete and timely administration of prophylactic medication and necessary supportive treatments may contribute to these results. Of note, after the first few cycles, the frequency of some side effects, such as pain or fever usually decreases. In the current study, hematological toxicities (Grade 3-4) including leukopenia and thrombocytopenia, that were possibly related to chemotherapy, were common, although they did not affect the treatment course. Furthermore, Grade 3-4 transaminase elevation was identified in one out of every four patients, leading to interruption of the treatment. The fact that severe hypertransaminasemia was not observed so frequently in our patients receiving maintenance DB suggests that temozolomide may have contributed to this condition. In general, immunotherapy-related toxicities were temporary and disappeared with the appropriate supportive care or discontinuation of antibody infusion.

Nonetheless, there are some limitations to this study. One limitation of this trial is the single referral center setting. The other limitations are the relatively small size of the cohort, retrospective design, short follow-up period and lack of comparison group.

## Conclusion

In conclusion, our study results suggest that DB-based chemo-immunotherapy seems to be suitable with encouraging response rates in patients with R/R HR-NB. Further large-scale, multi-center, prospective studies are warranted to confirm these preliminary results.

## Data availability statement

The original contributions presented in the study are included in the article/supplementary material. Further inquiries can be directed to the corresponding author.

## Author contributions

NO, EC and Dİ conceived of the presented idea and designed the study. All authors carried out the research. NO, EC and Dİ wrote the manuscript with input from all authors. All authors discussed the results and commented on the manuscript. All authors contributed to the article and approved the submitted version.
